# 7-α-O-Methylmorroniside ameliorated brain injury in 5×FAD mice by regulating the gut microbiome and NMDAR2B

**DOI:** 10.3389/fphar.2025.1545566

**Published:** 2025-05-30

**Authors:** Fengxiao Hao, Mengnan Zeng, Bing Cao, Xiwen Liang, Zhiyou Hao, Kaili Ye, Xinmian Jiao, Weisheng Feng, Xiaoke Zheng

**Affiliations:** ^1^ College of Pharmacy, Henan University of Chinese Medicine, Zhengzhou, China; ^2^ The Engineering and Technology Center for Chinese Medicine Development of Henan Province, Zhengzhou, China

**Keywords:** Alzheimer’s disease, 5×FAD mice, 7-α-O-Methylmorroniside, gut microbiome, NMDAR2B

## Abstract

Alzheimer’s disease (AD) is a neurodegenerative disorder characterized by cognitive decline. 7-α-O-Methylmorroniside (MorA), an iridoid glycoside extracted from *Cornus officinalis Sieb.* et Zucc., has been shown to have neuroprotective effects, but the mechanism of its anti-AD effect has not been clarified. In the present study, we investigated the mechanism by which MorA ameliorated brain injury in 5×FAD mice by using gut microbiota (GM) combined with *in vitro* and *in vivo* pharmacological experiments. Behavioral tests revealed that MorA could enhance learning and memory ability and improve cognitive impairment. The results of pathology, flow cytometry and biochemical indexes showed that MorA could reduce the levels of neuronal apoptosis, oxidative stress, Aβ_1-40_, Aβ_1-42_, p-Tau, and inflammatory factors in the mouse brain tissues, and improve brain damage. 16S rDNA sequencing showed that MorA increased the abundance of the beneficial bacterium *Lactobacillus* and decreased the abundance of the inflammation-associated Muribaculaceae and Prevotellaceae, and that these differential bacteria were closely associated with brain biochemical indicators. In addition, pathway enrichment analysis, Western blot and molecular docking results showed that the ameliorative effect of MorA on brain injury in 5×FAD mice was closely related to NMDAR2B. Next, an inhibitor of NMDAR2B was added to Aβ_25-35_-induced N9 and PC12 cells to further investigate whether the effect of MorA on AD was mediated through NMDAR2B. In conclusion, MorA ameliorated brain injury in 5×FAD mice by restoring GM homeostasis and inhibiting NMDAR2B.

## 1 Introduction

Alzheimer’s disease (AD) is a degenerative disease of the central nervous system, characterized by progressive cognitive deficit and behavioral impairment, which commonly occurs in elderly individuals (Rabbito et al., 2020). Symptoms such as emotional apathy, cognitive impairment and language deficits are common in people with AD. The primary pathological features of AD are plaques formed by the deposition of amyloid-beta (Aβ) protein and neurofibrillary tangles composed of phosphorylated tau proteins (Breijyeh and Karaman, 2020). Aβ deposition also contributes to neuronal death and brain damage by accelerating apoptosis and promoting oxidative stress and inflammation, ultimately exacerbating AD. In addition, cholinergic nerve damage, excitatory neurotoxicity, and mitochondrial dysfunction are also closely related to the development of AD (Richter et al., 2019; Yu et al., 2025; Swerdlow et al., 2014). Therefore, specifying the potential pathogenesis of AD is essential for its treatment.

In recent years, there has been increasing evidence that gut microbiota (GM) disorders are associated with AD (Wang, 2023). The enormous and diverse GM in the human body plays a crucial role in maintaining various molecular and cellular processes (Carloni and Rescigno, 2023) and can interact with the brain through a variety of pathways and processes, such as the vagus nerve and the immune system, to maintain brain homeostasis (Bano et al., 2024). Studies have shown that loss of GM not only increases the permeability of the gut and blood-brain barrier and activates the immune system (Mahadev et al., 2023), but also induces the release of large amounts of amyloids, lipopolysaccharides, and neurotoxins, increases pro-inflammatory mediators, and ultimately leads to neurodegeneration, which contributes to the process of AD (Nafady et al., 2022). Thus, sequencing of GM can be used as a tool for exploring the pathogenesis and therapeutic drugs of AD, which can be helpful for AD drug discovery.

7-α-O-Methylmorroniside (MorA) is an iridoid glycoside obtained from *Cornus officinalis Sieb*. et Zucc (Yang et al., 2022). It was shown to have significant neuroprotective activity against glutamate-induced toxicity in HT22 hippocampal cells (Jeong et al., 2012). However, there is no evidence demonstrating that MorA is effective in transgenic AD mouse models. Five gene loci with familial mutations in AD are present in transgenic 5×FAD mice (Song et al., 2021), and the results of most experimental and research analyses can now confirm that the mutated genes induce amyloid plaque formation, neurodegeneration, and behavioral dysfunction that are similar to those of AD patients (Ge et al., 2021). As a result, 5×FAD mice are commonly utilized to study AD-related pathologies and therapeutic approaches. Memantine (Mem) is a drug approved by the USA Food and Drug Administration (FDA) for the treatment of AD (Tang et al., 2023), which can exert anti AD effects by blocking N-methyl-D-aspartate receptors (NMDAR) and reducing calcium influx (Kabir et al., 2019). Therefore, we chose Mem as the positive control. In this study, we investigated the effect and mechanism of MorA on brain damage in 5×FAD mice using 16S rDNA sequencing and pharmacological experiments. This will provide an experimental basis for the medical value of MorA.

## 2 Materials and methods

### 2.1 Medicine

MorA was isolated from *C. officinalis Sieb*. et Zucc, purity ≥ 98% (Yang et al., 2022). Aβ_25-35_ peptides were purchased from Sangon Biotech Co., Ltd., dissolved in double-distilled water and aggregated by incubation at 37°C for 7 days. Memantine (CAS:41100-52-1, purity ≥ 98%) was purchased from Macklin Biochemical Technology Co., Ltd. (Shanghai, China).

### 2.2 Animals

**FIGURE 1:**
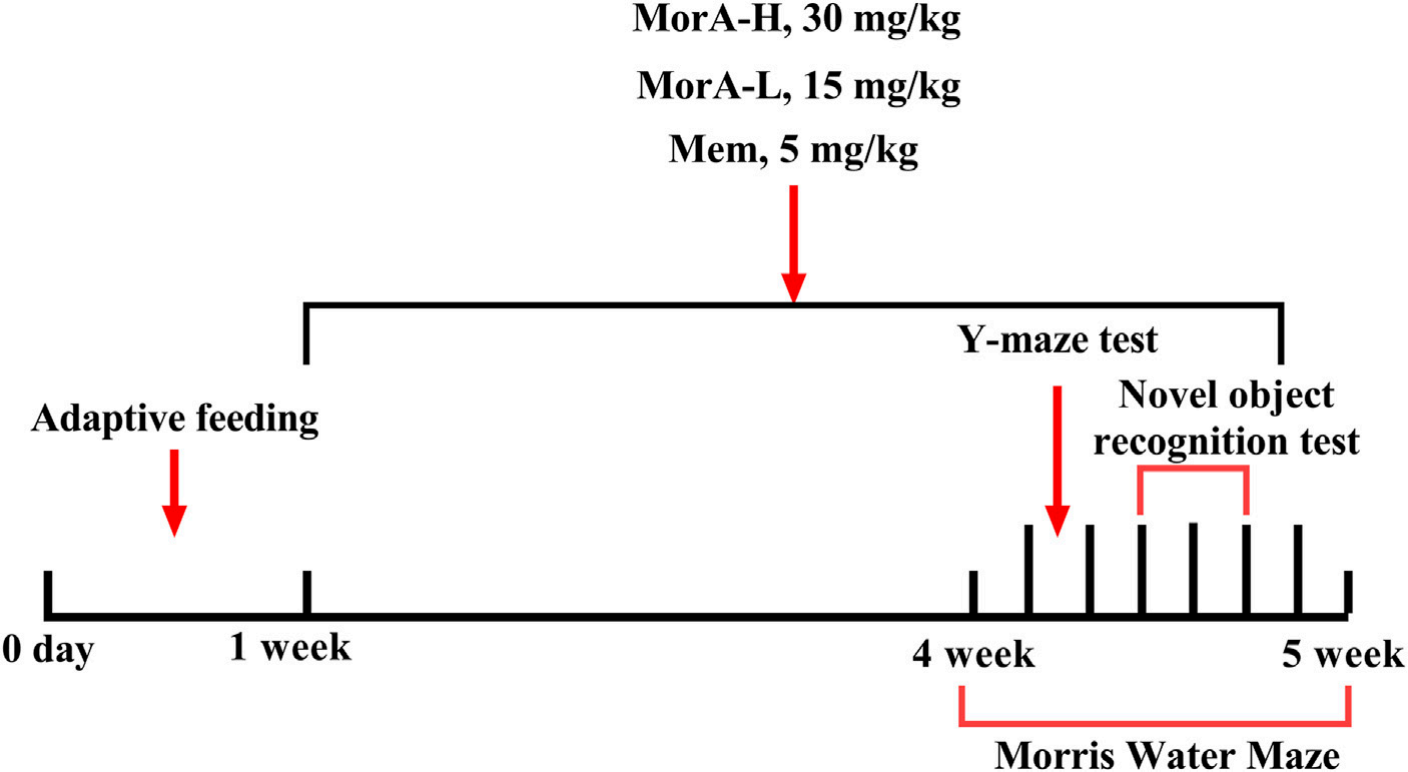
Process of experiment.

C57BL/6J mice (WT, 4–5 months old, n = 10) and 5×FAD mice (4–5 months, n = 40) were purchased from Shanghai Southern Model Biotechnology Co., Ltd. and kept in a clean-grade animal room at 18°C–24°C with a standard light/dark cycle. The mice had free access to water and food. Animal experiments were approved by the Ethics Committee of Henan University of Traditional Chinese Medicine (Ethics No. DWLL2018080003). After 1 week of acclimatization feeding, C57BL/6J mice were used as the control group (WT), while forty 5×FAD mice were randomly divided into four groups: model (M), memantine-treated (5 mg/kg, Mem), low-dose MorA-treated (15 mg/kg, MorA-L), and high-dose MorA-treated (30 mg/kg, MorA-H). Mem and MorA were then administered by gavage once a day for four consecutive weeks. The WT group and the M group were given the same amount of normal saline. Behavioral testing was performed 1 week before the animals were euthanized. The experimental procedure is shown in Figure 1.

### 2.3 Behavioral tests

#### 2.3.1 Y-maze test

The Y-maze alternation test is frequently used to investigate short-term memory (Kraeuter et al., 2019). As we previously reported, the total number of times the mice entered each arm (N) was recorded, with the successive entry into the three different arms being recorded as an alternation (N1) (Cao et al., 2021). The percentage of alternation (%) = [N1/(N-2)] × 100%.

#### 2.3.2 Novel object recognition test (NOR)

The NOR test assesses cognitive and memory abilities based on the innate preference of rodents for novelty (Da Cruz et al., 2020). The detection method was as previously described (Cao et al., 2021), we positioned two identical objects (A0 and A1) at the left and right ends of a square arena, respectively. The mouse was placed equidistant from both objects, and the time spent exploring the novel object was recorded during a 5-min session. After 24 h, A0 was replaced with a novel object B, and the exploration time toward object A1 (TA1) and novel object B (TB) was measured over another 5-min period. Then the preferential index = TB/(TA1+TB) and discrimination index = (TB-TA1)/(TA1+TB) were calculated (Zeng et al., 2022a).

#### 2.3.3 Morris water maze (MWM)

The MWM test is the most commonly used experimental method to evaluate spatial learning and memory. It can effectively assess hippocampus-dependent learning and memory (Othman et al., 2022). A 120-cm-diameter pool filled with milky white water and a video tracking system were used to perform the MWM test. The platform was placed in one quadrant of the pool, approximately 1 cm from the water surface. The experiment lasted 7 days. On the first day of the experiment, the mice were allowed to explore freely for 5 min, after which they were placed on the platform for 1 min. From the 2nd day to the 6 day of the experiment, the mice were placed into the water facing the wall of the pool and allowed to explore for 60 s. If the mice found the platform within 60 s, they could rest for 1 min; if they failed to find the platform, they were guided to it and stayed there for 1 min. On the 7 day of the experiment, the platform was removed, and the mice were placed in the water at one of the same entry points. The time spent in the original platform quadrant and the number of times the mice crossed the platform within 60 s were recorded. Trajectory acquisition was performed using the Animal Behavior Trajectory Video Analysis System V3.0 (Labmaze, Beijing Zhongshi Dichuang Technology Development Co., Ltd., China).

### 2.4 Haematoxylin-eosin (HE) staining

After the behavioral experiments were finished, mice were euthanized by cervical dislocation following isoflurane anesthesia. The brains of mice were rapidly dissected on ice and fixed in 4% paraformaldehyde solution for 24 h. Afterwards, they were embedded in paraffin and processed into 5 μm paraffin-embedded sections. Then, the sections were stained with HE. Images were captured using an OLYMPUS BX53F2 microscope.

### 2.5 Thioflavin S staining

Brain tissue sections were deparaffinized and washed with PBS, after which the processed sections were incubated with 0.5% thapsigargin S dissolved in 50% ethanol for 30 min. At the end of the incubation, the sections were differentiated twice with 80% ethanol and washed with distilled water. Finally, the stained sections were incubated in 5×PBS solution at 4°C for 1 h and blocked. Fluorescence images were scanned and obtained by Wuhan Servicebio Biotechnology Co., Ltd., screenshots were taken using CaseViewer software, and fluorescence intensity was quantified by ImageJ software.

### 2.6 Flow cytometry analysis of apoptosis, ROS and Ca^2+^ levels

After the mice were executed, the hippocampus was carefully isolated from the mouse brain and then gently crushed in 12 mL of sterile PBS. Then the samples were filtered through a 70 μm filter membrane and centrifuged at 1,500 rpm for 5 min. The supernatant was discarded to obtain the primary hippocampal cells of mice. Primary hippocampal cells were resuspended in 300 µL PBS, and the suspension was divided equally into three portions. Apoptosis, ROS and Ca^2+^ levels were detected using Annexin V/PI (BD Biosciences 556547, United States), DCFH-DA (CA1410; Solarbio, Beijing, China), and Fluo-4 AM (F8500, Solarbio, Beijing, China) kits, respectively, following the kit instructions. The level of apoptosis was measured and analyzed by flow cytometry (BD FACSAria III, United States), while ROS and Ca^2+^ were measured using Flowsight FlowSight^®^ (Luminex, Austin, Texas, United States).

N9 and PC12 cells were treated with drugs for 24 h and then centrifuged to obtain single cells. The cells were stained using the Annexin V/PI kit, DCFH-DA, and Fluo-4 AM, respectively. Cells were then collected using FACS Aria III and apoptosis, ROS and Ca^2+^ levels were determined.

### 2.7 Biochemical indexes

Take about 80 mg of mouse brain tissue and prepare a brain tissue homogenate with a concentration of 100 mg/mL using physiological saline, and the supernatant was centrifuged to measure the levels of mouse beta amyloid beta 1–42 (Aβ_1-42_, E-EL-M3010), mouse beta amyloid beta 1–40 (Aβ_1-40_, E-EL-M3009), phosphorylated Tau protein (p-Tau, E-EL-M1289c), mousetumor necrosis factor-α (TNF-α, E-EL-M0049c), mouse interleukin-6 (IL-6, MM-0163M1), mouse interleukin-1β (IL-1β, MM-0040M1), mouse glutathione (Glu, MM-44113M1), superoxide dismutase (SOD, A001-3-2, Nanjing Jiancheng Bioengineering Institute, Nanjing, China), glutathione peroxidase (GSH-Px, A005-1-2, Nanjing Jiancheng Bioengineering Institute, Nanjing, China), and malondialdehyde (MDA, A003-1-2, Nanjing Jiancheng Bioengineering Institute, Nanjing, China).

### 2.8 Gut microbiota (GM) analysis

Faecal samples were collected from the mice before dissection. The total DNA of the microbiome samples from the feces of 5×FAD mice was extracted by CTAB. Full-length 16S rDNA PCR was performed using primers: 27F: 5′-AGRGTTYGATYMTGGGCTCAG-3′ and 1492R: 5′-RGYTACCTTGTTACGACTT-3′. Next, Hangzhou Lianchuan Biotechnology Co., Ltd. carried out 16S rDNA sequencing using a PacBio Sequel II sequencer. Graphical visualization was performed using the Lianchuan Biotechnology Cloud Platform (Lyu et al., 2023).

### 2.9 Western blotting

Approximately 50 mg of mouse brain tissue was homogenized in lysis buffer, resulting in a final amount of 100 mg/mL. The supernatant was collected, the protein concentration was determined using a BCA Protein Extraction Kit (Solarbio, Life Science, Beijing, China), and the proteins were loaded into a gel using loading buffer. The sample size for each well is fixed at 30 μg and then the samples were separated by SDS‒PAGE, after which they were transferred to PVDF membranes. Next, 5% BSA was used for blocking. After blocking, primary antibodies N-methyl-D-aspartate receptor 2B (NMDAR2B, GW008516, 1:1,000) and β-Tubulin (I0094-I-AP, 1:1,000) were added and incubated overnight at 4°C, followed by incubation with secondary antibodies in the dark for 1 h. Imaging was performed using an Odyssey CLx infrared fluorescence scanning imaging system (Clx, Li-COR, United States) and analyzed using Image studio software.

### 2.10 Molecular docking

The PDB files of glutamate receptor protein NMDAR2B (PDB:7UJT) was downloaded from the Protein Data Bank database (https://www.rcsb.org/). The 2D structure of MorA was downloaded from the PubChem database (https://pubchem.ncbi.nlm.nih.gov/). Molecular docking was performed using the SailVina platform, after which the molecular docking results were exported using OpenBabel software and PyMOL software, and then the binding sites were identified using the protein–ligand interaction profiler (PLIP) website (https://projects.biotec.tu-dresden.de/plip-web/plip/index).

### 2.11 Cell culture

N9 microglial cells (purchased from Beina Biotechnology Co., Ltd.) were maintained in RPMI-1640 medium (Gibco, 31800-022, United States) with 10% fetal bovine serum (FBS, Hangzhou Four Seasons, 11011-8611) in a 37°C, 5% CO_2_ incubator, while highly differentiated PC12 cells (PC12) (purchased from Procell Life Science and Technology Co. Ltd.) were cultured in Dulbecco’s modified of Eagle’s medium (DMEM, Gibco, 12100-061, United States) containing 10% fetal bovine serum (FBS, Hangzhou Four Seasons, 11011-8611) in a 37°C, 5% CO_2_ incubator.

### 2.12 Cell viability assay

PC12 and N9 cells were inoculated into 96-well cell culture plates (CCP-96H, Wuhan Servicebio Biotechnology Co., Ltd., China) at 5 × 10^4^ and 3 × 10^4^ cells/well, respectively. Next, they were divided into the following groups: control group (CON); model group (M, Aβ_25-35_, 1 μM for PC12 cells, 0.1 μM for N9 cells); and MorA group (0.01, 0.05, 0.1, 0.2, 0.25, 0.5, 1, 2 μM). MTT solution (20 μL, 5 mg/mL) was added to each well after 24 h and then incubated at 37°C for 4 h. After discarding the medium, 150 µL DMSO was added, and the plate was shaken for 10 min. The OD value was then measured at a wavelength of 490 nm on an EPOCH microplate reader (BioTek, Winooski, VT, United States).

### 2.13 Effect of MorA on Aβ_25-35_-induced cell damage

N9 and PC12 cells were seeded in six-well plates at densities of 6 × 10^4^ and 1 × 10^5^ cells per well, respectively. They were divided into six groups: the normal group (CON), model group (M, Aβ_25-35_, 1 μM for PC12 cells, 0.1 μM for N9 cells), MorA group (0.2 μM for PC12 cells, 0.5 μM for N9 cells, Aβ_25-35_, 0.1 μM or 1 μM), and groups with NMDAR2B inhibitor (MK-801, 10 μM) added separately. MK-801 was administered 1 h before the start of drug treatment, and the cells were collected 24 h later. Apoptosis, ROS and Ca^2+^ levels were then determined using FACS Aria III (BD Biosciences, United States).

### 2.14 Statistical analysis

Experimental data were analyzed using IBM SPSS 26.0 software and Tukey test was used to compare the differences between groups. P values less than 0.05 was considered significant.

## 3 Results

### 3.1 MorA enhances learning memory and ameliorated cognitive deficits in 5×FAD mice

**FIGURE 2:**
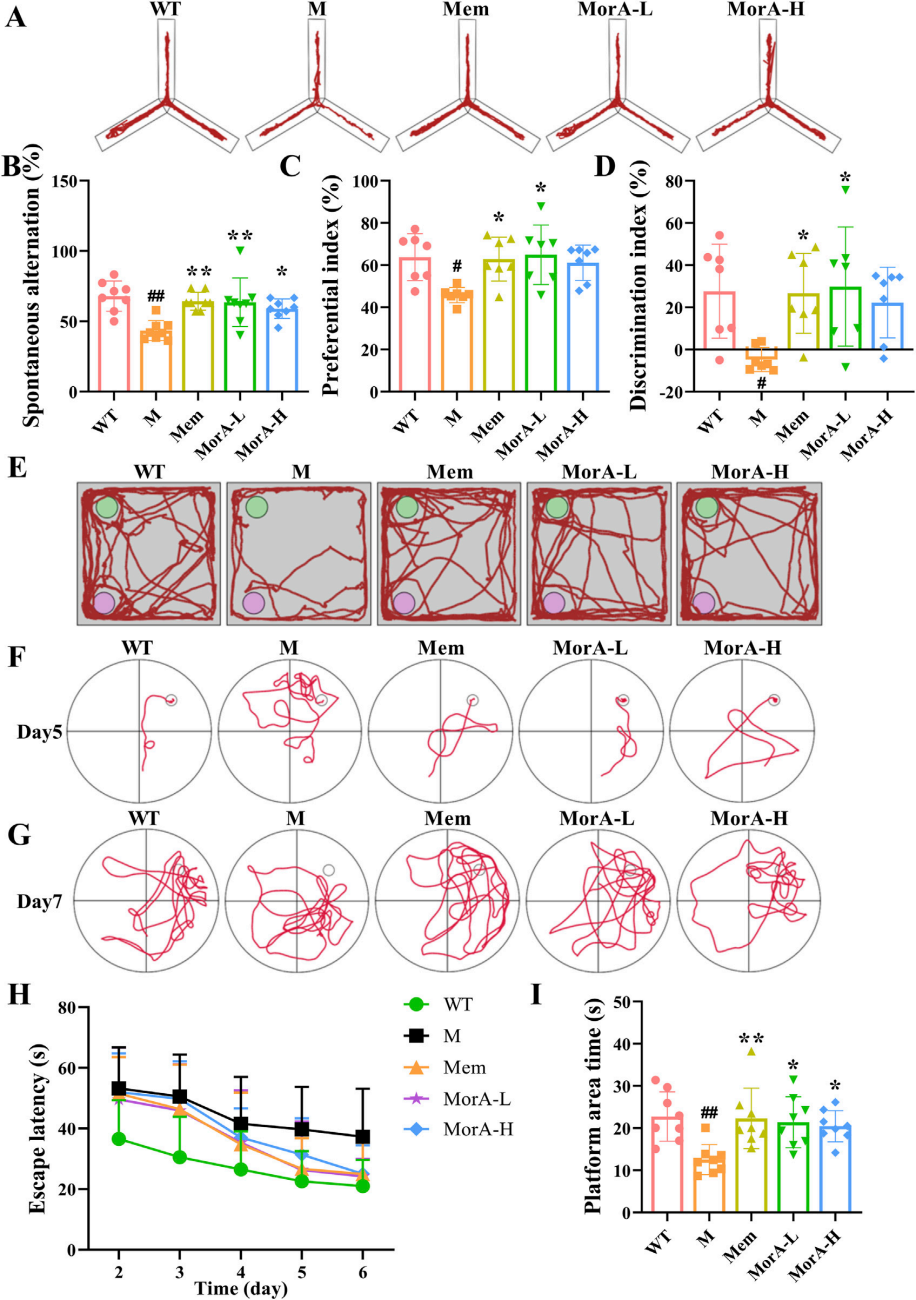
Effects of MorA on learning memory and cognitive deficits in 5×FAD mice. **(A, B)** Y-maze trajectory maps and quantitative result of spontaneous alternation rate. **(C–E)** NOR trajectory maps and quantitative result of preference and discrimination index **(F–I)** Representative images of MWM and quantification of escape latency and residence time in the platform area. n = 8, ^#^ *p* < 0.05, ^##^ *p* < 0.01 vs. WT; *p < 0.05, **p < 0.01 vs. M.

To assess the effect of MorA on the amelioration of learning memory and cognitive ability in 5×FAD mice, we performed behavioral tests. The Y-maze results showed that the spontaneous alternation rate was significantly lower in 5×FAD mice (*P* < 0.01), while the spontaneous alternation rate in the Mem, MorA-L, MorA-H groups were increased (*P* < 0.01 or *P* < 0.05, Figures 2A, B). The results of the NOR test showed that the Mem and MorA-L groups significantly increased the preference index and discrimination index of new object in 5×FAD mice compared with the M group (*P* < 0.05, Figures 2C–E). The MWM was used to further investigate the effect of MorA on learning memory, and the results showed that the escape latency of mice in all groups were greatly shortened from 2 to 6 days (Figures 2F, H), and mice in the Mem, MorA-L, and MorA-H groups stayed in the platform area for a longer period of time after the platform was removed (*P* < 0.01 or *P* < 0.05, Figures 2G, I). From these results, it can be observed that Mem, MorA-L and MorA-H improved learning memory ability and cognitive deficits in 5×FAD mice, in which the treatment effect of MorA-L is better than that of MorA-H. Therefore, only the results of MorA-L were shown in the subsequent experiments.

### 3.2 MorA attenuated pathological brain damage in 5×FAD mice

**FIGURE 3:**
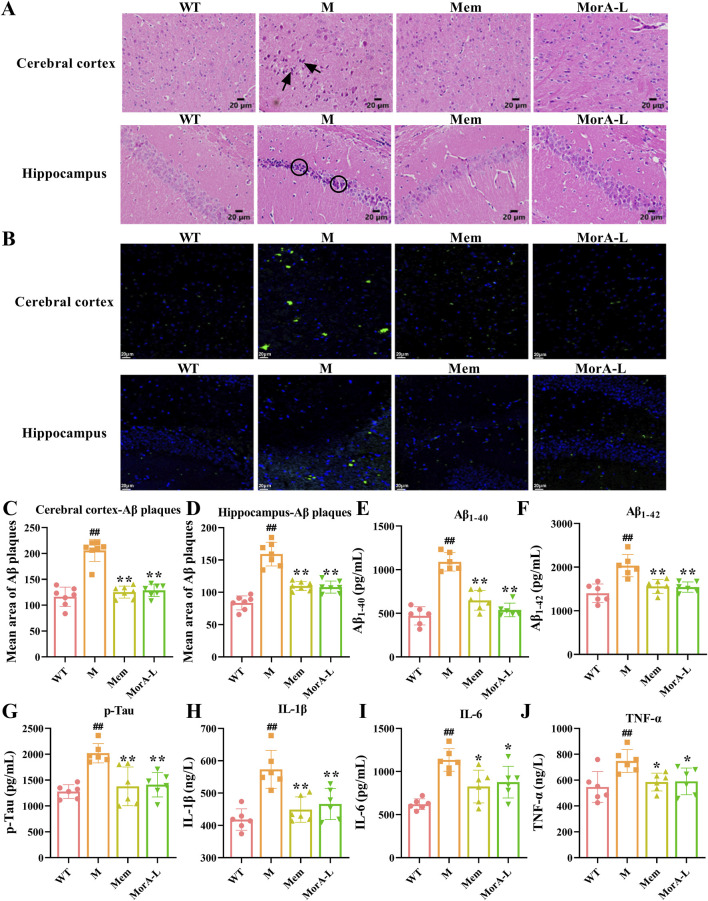
Effects of MorA on pathological brain damage in 5×FAD mice. **(A)** Characteristic pictures of HE staining (×400, scale bar = 20 μm, the arrows and circles in the figure are damaged nerve cells). **(B–D)** Representative images and quantitative results of Aβ plaques (×400, scale bar = 20 µm). **(E–G)** Quantification of Aβ_1-40_, Aβ_1-42_ and p-Tau levels in brain tissue of 5×FAD mice. **(H–J)** Quantitative analysis of IL-1β, IL-6 and TNF-α levels in brain tissues of 5×FAD mice. n = 6. ^##^ *p* < 0.01 vs. WT; *p < 0.05, **p < 0.01 vs. M.

The H&E staining results showed that neurons in the cortical and hippocampal regions of mice in the M group exhibited deepened staining, cell atrophy, and a decrease in the number of normal cells, whereas neuronal cells in mice treated with Mem and MorA demonstrated normal morphology, neat arrangement, and typical neural texture (Figure 3A). Then, Aβ plaques in the brain tissue of mice were observed by thioflavine staining and it was found that the area of Aβ plaques in the brain tissue of mice in the M group was significantly increased, and there were different degrees of improvement after the administration of Mem and MorA interventions (*P* < 0.01, Figures 3B-D). In addition, detection of characteristic pathological indicators revealed that Mem and MorA significantly reduced the levels of Aβ_1-40_, Aβ_1-42_, and p-Tau in the brain tissues of 5×FAD mice (*P* < 0.01, Figures 3E–G), as well as the levels of inflammatory factors IL-1β, IL-6, and TNF-α (*P* < 0.01 or *P* < 0.05, Figures 3H–J). These results suggested that MorA attenuated pathological damage in brain tissue of 5×FAD mice and that its effect is comparable to that of Mem.

### 3.3 MorA reduced apoptosis, oxidative stress and Ca^2+^ levels in the brain tissue of 5×FAD mice

**FIGURE 4:**
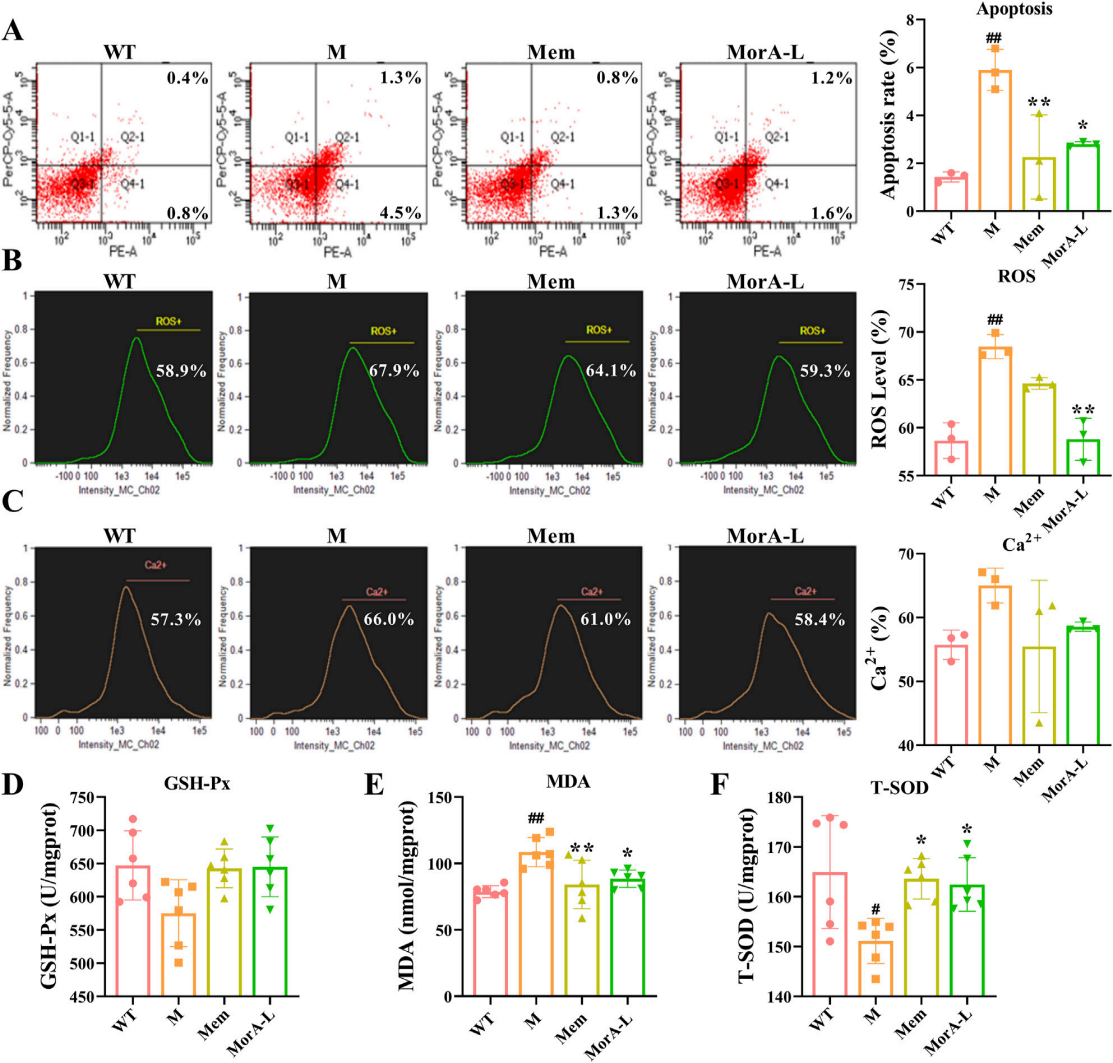
Effect of MorA on apoptosis, oxidative stress and Ca^2+^ levels in the brain tissue of 5×FAD mice. **(A)** Representative images and quantitative results of apoptosis levels in brain tissue of 5×FAD mice (Q1 represents mechanically damaged or nonspecifically dead cells, Q2 indicates late apoptotic cells, Q3 denotes viable cells, and Q4 corresponds to early apoptotic cells). **(B)** Representative images and quantitative analysis of ROS levels in brain tissue of 5×FAD mice. **(C)** Representative images and quantification of Ca^2+^ levels in brain tissue of 5×FAD mice. **(D–F)** Quantification of GSH-Px, MDA and T-SOD levels in brain tissue of 5×FAD mice. n = 3 or 6, ^#^ *p* < 0.05, ^##^ *p* < 0.01 vs. WT; *p < 0.05, **p < 0.01 vs. M.

We used flow cytometry to detect indicators of apoptosis and oxidative stress in mouse brain tissue to further explore the effects of MorA on brain injury in 5×FAD mice. The results indicated that the apoptosis rate and ROS generation rate were significantly increased in the brain tissue of mice in group M. These indicators decreased significantly after treatment with Mem and MorA (*P* < 0.01 or *P* < 0.05, Figures 4A–C). Although the level of Ca^2+^ in mouse brain tissue was improved, it did not show significant difference. Moreover, Mem and MorA significantly reduced MDA levels and increased the expression of T-SOD in brain tissues of 5×FAD mice (*P* < 0.01 or *P* < 0.05, Figures 4E, F), but had no significant effect on GSH-Px (Figure 4D). The results in this section suggested that MorA ameliorated brain injury by reducing apoptosis, oxidative stress and Ca^2+^ levels in the brain tissue of 5×FAD mice.

### 3.4 MorA restored gut microbial imbalance in 5×FAD mice

**FIGURE 5:**
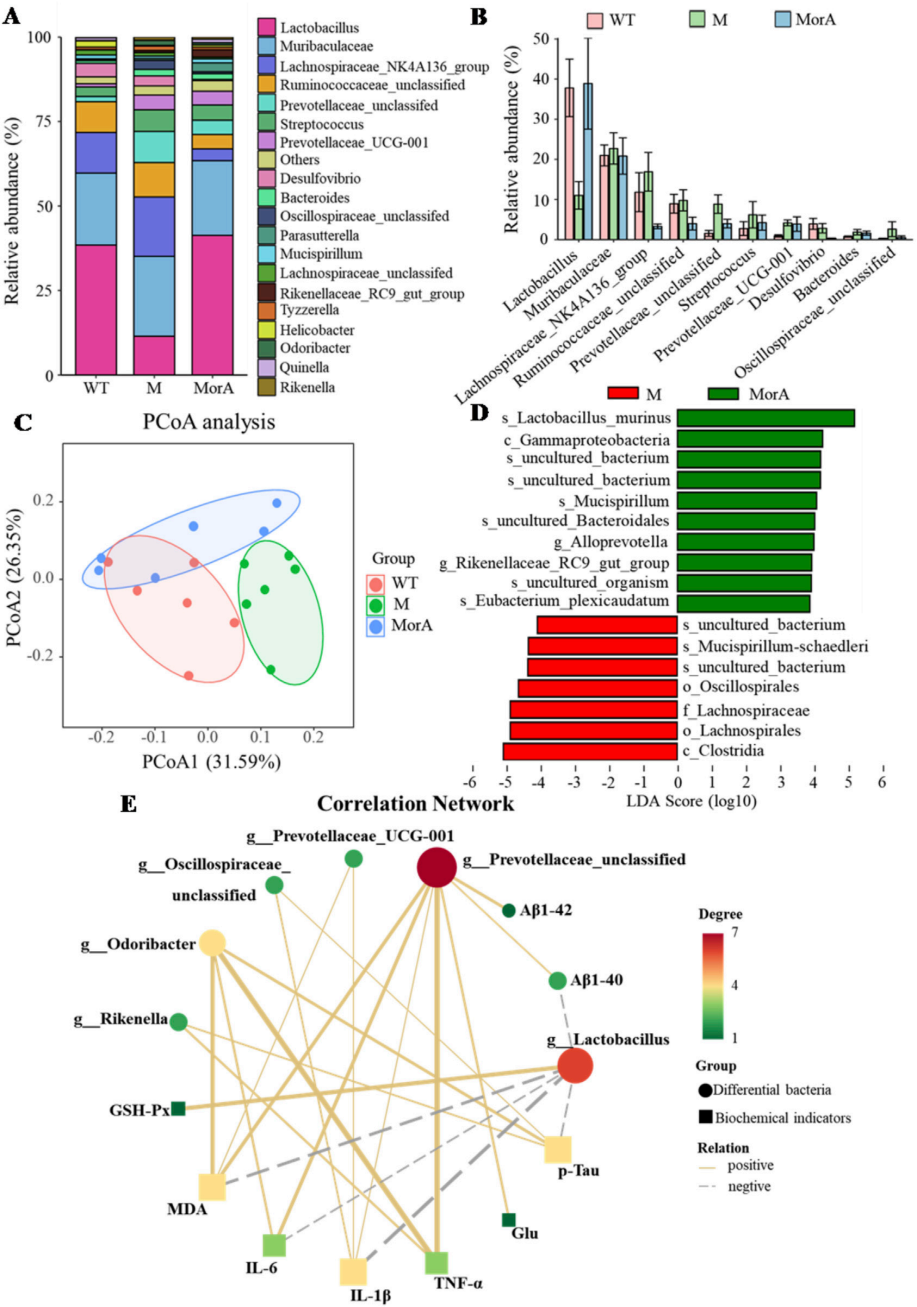
Effect of MorA on the GM in 5×FAD mice. **(A)** The 20 most abundant bacteria in feces. **(B)** Histogram of abundance changes. **(C)** PCoA. **(D)** LEfSe analysis. **(E)** KEGG pathway analysis. n = 6.

Alterations in the GM are closely related to the development of disease. Through 16S rDNA sequencing, it was possible to assess the composition of GM and explore changes in the GM of 5×FAD mice during MorA treatment. The top 20 most enriched genus-level groups in the GM are shown in Figure 5A. *Lactobacillus* abundance was increased, while Muribaculaceae, Lachnospiraceae_NK4A136, Ruminococcaceae, Prevotellaceae, and *Streptococcus* abundance were decreased after MorA treatment (Figure 5B). Principal coordinate analysis (PCoA) based on UniFrac distances showed significant characteristic clustering of the microbiota in the WT, M, and MorA groups (Figure 5C). LEfSe analysis revealed significant differences in microbial taxa between the model and MorA groups when the LDA score exceeded 3. (Figure 5D).

### 3.5 Interaction of GM with brain biochemical indices

The correlation analysis of the top 20 genus-level microbe abundance in the GM with the brain biochemical indices (Figure 5E) showed that *Lactobacillus* was significantly negatively correlated with MDA, IL-6, IL-1β, p-Tau, and Aβ_1-40_ levels and Prevotellaceae was significantly negatively correlated with Aβ_1-40_, Aβ_1-42_, Glu, MDA, IL-6, TNF-α, and IL-1β levels. Prevotellaceae_UCG-001 showed a positive correlation with MDA and IL-1β levels and Oscillospiraceae showed a positive correlation with IL-1β and p-Tau levels. Finally, Odoribacter was positively correlated with MDA, IL-6, TNF-α, and p-Tau levels, and Rikenella was positively correlated with TNF-α and p-Tau levels.

### 3.6 MorA regulated NMDAR2B in the brain of 5×FAD mice

**FIGURE 6:**
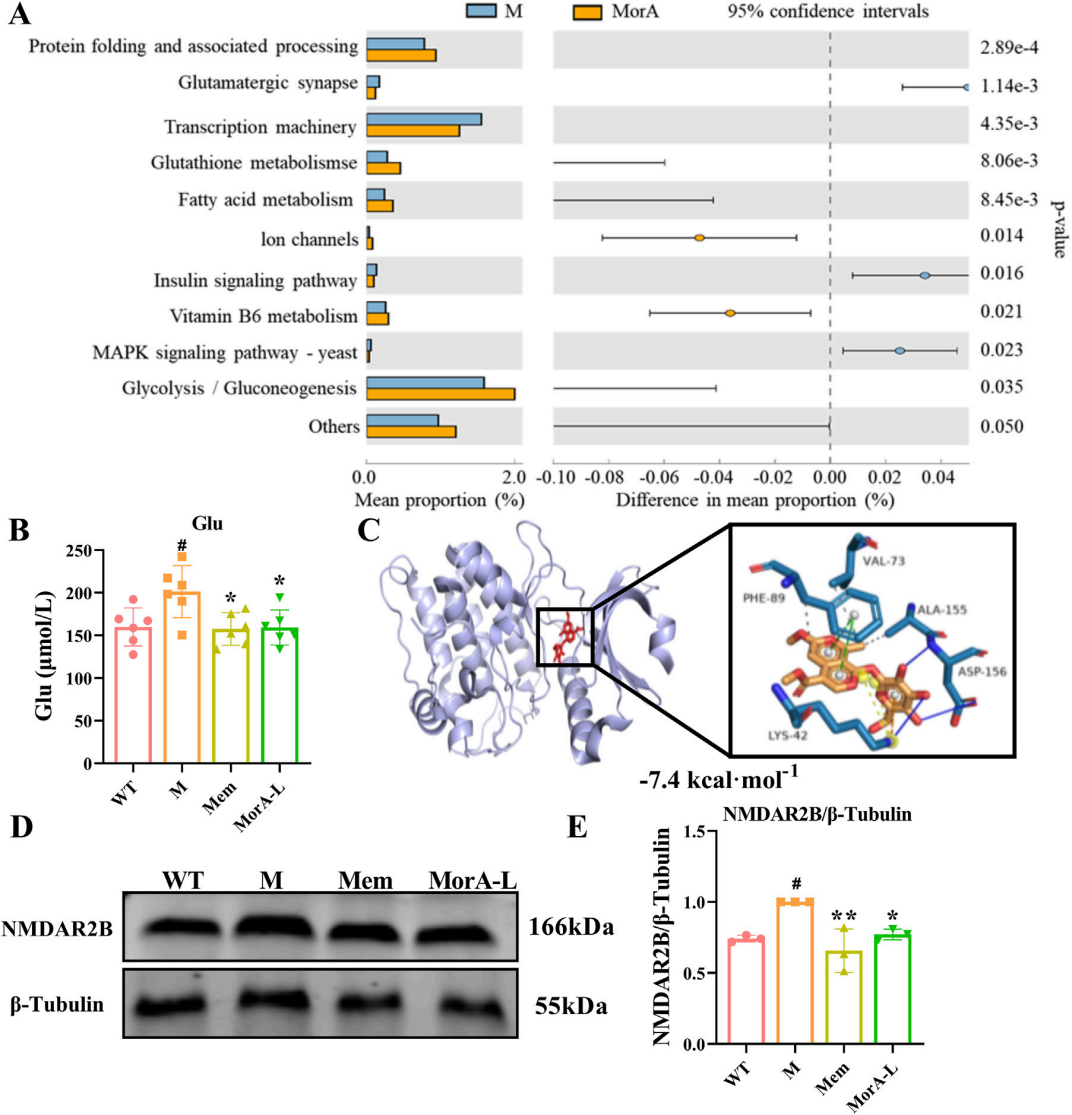
Effect of MorA on NMDAR2B in the brain of 5×FAD mice. **(A)** PICRUSt functional prediction. **(B)** Quantification of Glu level in brain tissue of 5×FAD mice. **(C)** Molecular docking results of MorA with NMDAR2B. **(D, E)** Representative WB images and quantitative analysis of the protein expressions of NMDAR2B. n = 3 or 6, ^#^ *p* < 0.05 vs. WT; *p < 0.05, **p < 0.01 vs. M.

PICRUSt functional predictions showed that the therapeutic effects of MorA on AD involve glutamatergic synapse, transcription machinery, glutathione metabolism, and so on (Figure 6A). Glutamate is a class of excitatory neurotransmitters *in vivo*, and its abnormalities can lead to neurotoxicity. Our examination of glutamate levels in mouse brain tissue revealed an abnormal increase in glutamate levels in brain tissue of 5×FAD mice (*P* < 0.05, Figure 6B), suggesting possible excitotoxicity. Glutamate-induced excitotoxicity is closely related to the glutamate receptor NMDAR2B. To further investigate whether MorA exerts therapeutic effects by affecting NMDAR2B, we modeled the binding mode of MorA and NMDAR2B by molecular docking and found that there are three hydrophobic interactions, three hydrogen bonds and two π-stacking interactions between MorA and NMDAR2B (Figure 6C). Furthermore, we found that NMDAR2B protein levels were also significantly increased in 5×FAD mice, and MorA inhibited NMDAR2B expression (*P* < 0.05, Figures 6D-E). The above results indicated that MorA regulated NMDAR2B in the brain of 5×FAD mice.

### 3.7 Inhibition of NMDAR2B reversed the ameliorative effect of MorA on Aβ_25-35_-induced N9 and PC12 cells damage

**FIGURE 7:**
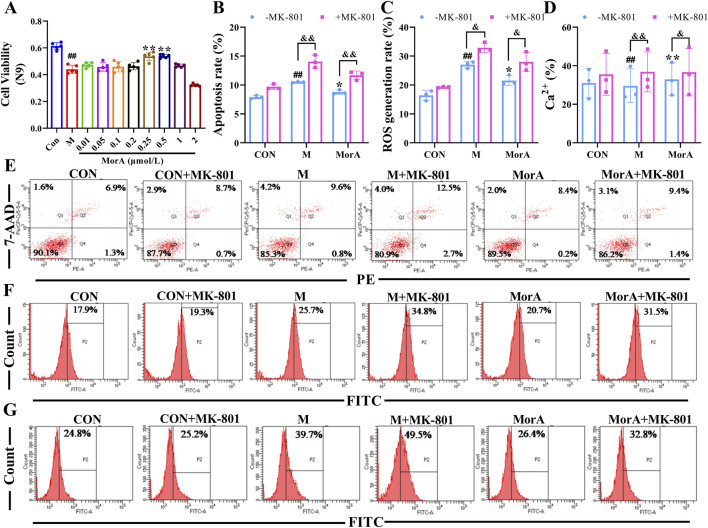
Effect of MorA on apoptosis, ROS, and Ca^2+^ in N9 cells. **(A)** The viability of N9 cells. **(B–D)** Quantification of the apoptosis, ROS and Ca^2+^ levels in N9 cells. **(E–G)** Representative images of apoptosis, ROS, and Ca^2+^ levels in N9 cells detected by flow cytometry. n = 3. ^##^ *p* < 0.01 vs. CON; *p < 0.05, **p < 0.01 vs. M. ^&^ *p* < 0.05, ^&&^ *p* < 0.01 vs. -MK-801.

**FIGURE 8:**
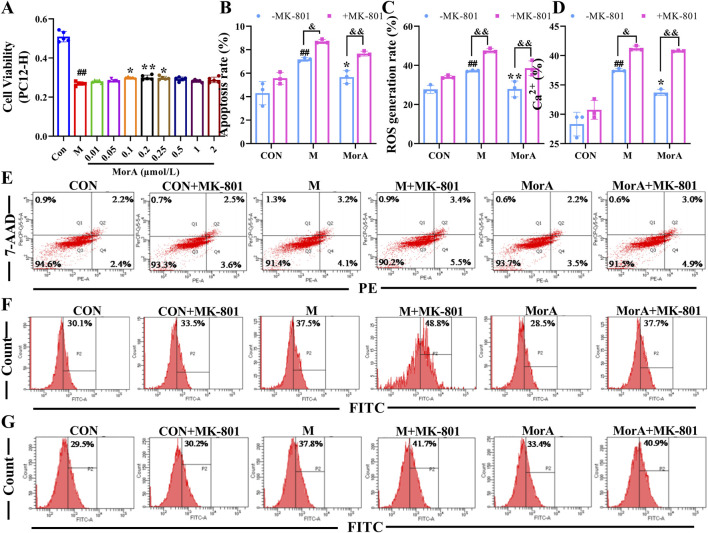
Effect of MorA on apoptosis, ROS, and Ca^2+^ in PC12 cells. **(A)** The viability of PC12 cells. **(B–D)** Quantification of the apoptosis, ROS and Ca^2+^ levels in PC12 cells. **(E–G)** Representative images of apoptosis, ROS, and Ca^2+^ levels in PC12 cells detected by flow cytometry. n = 3. ^##^ *p* < 0.01 vs. CON; *p < 0.05, **p < 0.01 vs. M; ^&^ *p* < 0.05, ^&&^ *p* < 0.01 vs. -MK-801.

To further investigate whether MorA exerts its therapeutic effect through NMDAR2B, Aβ_25-35_ was used to induce cell injury in N9 and PC12 cells, and apoptosis, ROS and Ca^2+^ were measured. The MTT results showed that the cell viability of N9 and PC12 cells were significantly reduced after incubation with Aβ_25-35_ for 24 h. MorA maximized N9 cells viability at a concentration of 0.5 μM (*P* < 0.01, Figure 7A) and significantly increased PC12 cells viability at a concentration of 0.2 μM (*P* < 0.01, Figure 8A). Therefore, we used these as therapeutic doses in subsequent studies. Flow cytometry demonstrated that Aβ_25-35_ significantly increased apoptosis, ROS, and Ca^2+^ levels in N9 and PC-12 cells. In N9 (*P* < 0.01 or *P* < 0.05, Figures 7B-G) and PC12 (*P* < 0.01 or *P* < 0.05, Figures 8B-G) cells, MorA decreased Aβ_25-35_-induced apoptosis, ROS, and Ca^2+^ levels to varying degrees. However, the presence of MK-801 inhibited these effects of MorA.

## 4 Discussion

AD is the most common type of dementia and a 2023 report showed that 6.7 million Americans aged 65 and older currently have AD, which is expected to reach 13.8 million by 2060 (Rostagno, 2022; Alzheimers Dement, 2023). Continuously increasing morbidity, mortality, and healthcare costs are placing an enormous burden on families and society. Therefore, there is an urgent need to develop an effective treatment modality for AD.

The most prominent feature in the development of AD is an irreversible decline in memory and cognitive functioning (Portelius et al., 2015), which ultimately leads to emotional apathy and behavioral changes. Behavioral experiments provide an important basis for studying emotions and cognitive functions of the brain, and are often used to assess learning memory and cognitive abilities in mice (Araujo et al., 2021; Stover et al., 2015). The Y-maze, NOR, and MWM experiments illustrated that MorA improved learning memory in 5×FAD mice. In addition to memory impairment, imbalance of Aβ production and clearance in the brain leading to Aβ deposition to form senile plaques (SP) is one of the major pathologies of AD (Nilsson and Saido, 2014). Amyloid precursor protein (APP) is broken down by α- and γ-secretase into nontoxic fragments under physiological conditions and by β-secretase and γ-secretase under pathological conditions into fragments such as the toxic Aβ_1-42_ and Aβ_1-40_ (Chuang et al., 2021), which accumulate to eventually form age spots. Some studies have shown that a large amount of Aβ aggregates promote Tau protein phosphorylation, accelerating the formation of neurofibrillary tangles and triggering neuronal toxicity (Lee et al., 2022). In addition, Aβ deposition leads to the excessive production of oxygen free radicals and oxidative stress, which ultimately leads to neuronal apoptosis and neuronal necrosis (Eratne et al., 2018; Wu et al., 2018). Our study showed that MorA attenuated neuronal cell atrophy and apoptotic cell number, reduced Aβ plaque formation, and decreased the level of oxidative stress. Thus, MorA ameliorates neurotoxicity and oxidative stress induced by Aβ deposition in the brain tissue of 5×FAD mice.

In addition to Aβ deposition and oxidative stress, the role of the GM in AD has attracted widespread attention (Goyal et al., 2021). There are as many as 100 trillion microorganisms in the human body, and most of them reside in the gastrointestinal tract (Honda and Littman, 2012). It was noted that altered flora composition and dysbiosis were observed in both AD patients and animal models, and that they were strongly associated with Aβ plaque deposition and neuroinflammation (Pasupalak et al., 2024). The use of 16S rDNA sequencing can show changes in flora abundance and to some extent reflect the disease process. 16S rDNA detected differential species in the GM of mice in the model and MorA groups, and MorA increased the abundance of the beneficial bacterium *Lactobacillus* in 5×FAD mice, while the abundance of *Muribaculaceae*, *Lachnospiraceae_NK4A136*, *Ruminococcaceae*, *Prevotellaceae*, and *Streptococcus* decreased. In addition, GM imbalance leads to an imbalance in host immune regulation inducing an inflammatory response, disrupting gut barrier and blood-brain barrier function, and exacerbating the AD process (Cattaneo et al., 2017). The results of the correlation analysis between GM and biochemical indicators demonstrated a significant correlation between GM abundance and the levels of inflammatory factors and oxidative stress-related indicators. Thus, MorA improved AD by modulating the GM.

It has also been demonstrated that Gut microbes can affect the synthesis and release of neurotransmitters such as glutamate and γ-aminobutyric acid (Clarke et al., 2014) and participate in the pathogenesis of AD by influencing brain activity and development through the gut–brain axis (Wang and Kasper, 2014). We examined glutamate levels in the brain and found that they were significantly elevated in 5×FAD mice. And pathway prediction showed that MorA treatment of AD involves glutamatergic synapses. Glutamate is an important excitatory neurotransmitter in the brain (Wang et al., 2020) and acts mainly on NMDAR. NMDAR is predominantly found in the postsynaptic membrane of glutamatergic neurons. Its interaction with postsynaptic density protein 95 (PSD95) mediates downstream excitotoxicity (Chiu et al., 2019). NMDAR2B, a NMDAR subtype, is primarily involved in synaptic plasticity and learning memory (Maher et al., 2014). When glutamate levels are elevated in the brain, they overstimulate NMDAR2B, leading to increased Ca^2+^ influx, which induces neuroexcitotoxicity (Findley et al., 2019). We found that NMDAR2B was overactivated by abnormal glutamate levels in 5×FAD mice and that MorA, which binds tightly to NMDAR2B, inhibits NMDAR2B activation. To further investigate whether MorA exerts anti-AD effects via the NMDAR2B, we used N9 and PC12-H cells for *in vitro* validation with the addition of MK-801, which blocks the action of NMDAR2B (Zeng et al., 2022b). Among the Aβ fragments, Aβ_25-35_ is the shortest fragment that retains the toxicity of the full-length Aβ (1-40/42) peptide and induces AD (Guo et al., 2022). Therefore, we chose Aβ_25-35_ to construct the *in vitro* AD model. The results showed that the ameliorative effect of MorA on Aβ_25-35_-induced cell damage was reversed by MK-801. The above results suggest that MorA can attenuate brain damage by inhibiting NMDAR2B and attenuating glutamate-induced excitatory neurotoxicity.

## 5 Conclusion

MorA reduces neurotoxicity and ameliorates learning memory deficits and brain damage in 5×FAD mice by modulating GM and inhibiting NMDAR2B. This research indicates that MorA may be a potential drug for the treatment of AD, and the findings of this study have important implications for the development of Cornus officinalis Sieb. et Zucc. However, there are some limitations in this study. From the results, it can be seen that the effect of MorA at 15 mg/kg is better than that of 30 mg/kg (Figure 2), which suggests that we need to further study the efficacy and toxicological mechanism of MorA.

## Data Availability

The original contributions presented in the study are included in the article/Supplementary Material, further inquiries can be directed to the corresponding author.

## Abbreviations

AD, Alzheimer’s disease; GM, gut microbiome; MorA, 7-α-O-Methylmonoside; HE, hematoxylin-eosin; NOR, novel object recognition test; MWM, morris water naze; Aβ_1−42_, beta amyloid beta 1–42; Aβ_1−40_, beta amyloid beta 1–40; p-Tau, phosphorylated Tau protein; Glu, glutathione; SOD, superoxide dismutase; GSH-Px, glutathione peroxidase; MDA, malondialdehyde; TNF-α, tumor necrosis factor alpha; IL-6, Interleukin 6; IL-1β, Interleukin 1 beta; APP, β-amyloid precursor protein; NMDAR2B, N-methyl-D-aspartate receptor subunit 2B; PSD95, postsynaptic density protein 95; PVDF, polyvinylidene fluoride; DMEM, Dulbecco’s modified Eagle’s medium; DMSO, dimethyl sulfoxide; MTT, methyl thiazolyl tetrazolium; SP, senile plaques.
